# Posturographic sensory ratios provide evidence for neuroplasticity after computerized vestibular rehabilitation therapy in a single group interventional trial

**DOI:** 10.1186/s12984-025-01608-w

**Published:** 2025-04-11

**Authors:** Eytan A. David, Navid Shahnaz

**Affiliations:** 1 Otology, Neurotology, Skull Base Surgery, Clinical Instructor, Department of Surgery, University of British Columbia, Vancouver, Canada; 2 Audiology and Speech Sciences, University of British Columbia, Vancouver, Canada

**Keywords:** Vestibular, Balance, Neuroplasticity, Inner ear, Posture, Neuronal plasticity, Saccule and utricle

## Abstract

**Background:**

Vestibular deficits are common and debilitating. Many patients do not achieve satisfactory resolution of their symptoms with standard rehabilitation techniques. This study seeks to measure changes in computerized dynamic posturography sensory ratio information after computerized vestibular retraining therapy (CVRT).

**Methods:**

This prospective, single group, interventional study enrolled adult participants with stable, unilateral vestibular deficits. Before and after twelve twice weekly sessions of CVRT, and 4–6 and 10–12 months post-treatment, participants completed the Sensory Organization Test, from which sensory ratios (somatosensory - SOM, visual - VIS, vestibular - VEST, and visual preference - PREF) were calculated.

**Results:**

13 participants completed the intervention and post-retraining assessment; 9 completed the 4–6 and 10–12 month assessments. After CVRT, VIS increased by 11.6 (1.6 to 21.7) and VEST increased by 9.5 (0.6 to 18.3) and both remained significantly above baseline 10–12 months after treatment. The SOM and PREF ratios changed negligibly. Participants with mild disability (DHI ≤ 30) showed no change while participants with moderate-to-severe disability (DHI > 30) had significantly greater improvements in VIS (*P* = 0.0006) and VEST (*P* = 0.02) across all three post-treatment assessments.

**Conclusions:**

CVRT was associated with durable improvement in VIS and VEST sensory ratios and improved postural control under conditions that favour use of vestibular information, consistent with increased weighting of vestibular information over vision.

**Trial registration:**

Clinicaltrials.gov registration NCT04875013; 04/27/2021.

## Introduction

Vestibular dysfunction can result from absent, corrupted, or asymmetric vestibular afferents. In the days after vestibular loss, patients typically experience nausea, rotary hallucination, and imbalance. Acute static symptoms often resolve quickly, through modulation of neuronal activity in the vestibular nuclei, but many individuals experience long term dynamic symptoms, including dizziness and postural unsteadiness, due to impaired coordination of vestibular reflexes [1–3].

Standard vestibular therapy (SVT) exercises seek to promote compensation by one or more of three mechanisms: habituation, adaptation, and substitution [4]. Habituation exercises involve movements that provoke symptoms (i.e., nodding/shaking of the head or multi-axis movement). Adaptation exercises seek to promote long-term changes to ameliorate retinal slip and improve visual acuity by promoting calibrating of the vestibular ocular reflex or by behavioural strategies, such as anticipatory saccades [5]. Substitution exercises seek to train the brain to find alternative strategies, to estimate corrective ocular and musculoskeletal movement in order to maintain visual acuity and postural stability. While there is evidence for the efficacy of these exercises, many patients do not benefit [5, 6] and the efficacy of SVT for ameliorating dynamic postural instability is limited.

Exercises involving congruent stimuli– when vision, somatosensation, and gravity/acceleration cues from the vestibular organs are all in agreement - are effective for promoting habituation and substitution; however, conflicting stimuli may be important for compensation of dynamic movements. Telescopic glasses and head mounted displays with visual scenes that are decoupled from head motion have been shown to modulate vestibular ocular reflex (VOR) gain [7, 8]. Virtual reality interventions designed to elicit visual-vestibular conflict can reduce visual dependence of postural control in healthy individuals [9]. Likewise, tilting platforms that introduce conflict between somatosensory information from the feet and ankles and vision and gravity/acceleration sensors may be superior to standard vestibular exercises for both subjective outcomes and gait [10]. Conversely, optokinetic stimulation without specific training for sensory conflict increased visual dependence and susceptibility to postural instability given conflicting visual information. This same study found that a CDP-based intervention resulted in improved postural stability [11].

Individuals with unilateral deficits may rely on somatosensory or visual cues in order to maintain their balance. Such patients have intact vestibular function on the contralateral side and, depending on the offending lesion, may have some residual function on the affected side; however, many individuals struggle to use their intact function to achieve effective compensation and continue to suffer impaired dynamic balance [12].

The sensory organization test (SOT) may give insight to the compensation strategies used by individuals with vestibular deficits and show whether these strategies change with treatment. The SOT employs precise posturographic measurement of sway while participants attempt to maintain their balance during six different testing conditions. These conditions challenge the participant to maintain equilibrium while systematically removing or creating sensory conflict with the somatosensory and visual frames of reference. Disruption of visual and somatosensory information is accomplished by “sway referencing”– that is, tilting of the support surface and/or visual field such that their orientation remains constant in relation to the sway angle [13]. In this way, as the participant leans in the anteroposterior axis, there is no visual and/or somatosensory cue of their change in angle relative to vertical.

Analysis of a participant’s performance in these conditions can be used to gain understanding of how sensory information is integrated and weighted during maintenance of postural stability. The sensory ratios that form part of the standardized SOT (Table 1) offer insight into the participants’ sensitivity to loss of sensory input and weighting of these inputs [13]. The SOM (somatosensory) ratio estimates the decrement in postural control when the three sources of sensory input– visual, somatosensory, and vestibular– are reduced to two by closing of the eyes. The VIS (visual) ratio estimates the decrement when somatosensory information is lost through activation of the sway-referenced platform, leaving only visual and vestibular senses. The VEST (vestibular) ratio ‘isolates’ the vestibular sense through activation of the sway-referenced platform and by closing of the eyes. Finally, the PREF (visual preference) ratio, compares postural stability when participants are provided with conflicting, sway-referenced, visual information to performance with absent visual information (eyes closed).

We have previously reported durable improvement in patient-reported disability and objective posturography for patients with stable unilateral vestibular deficits [14, 15]. In this report, participants received computerized vestibular retraining therapy (CVRT), which challenges participants by systematic disruption of somatosensory and/or visual input or presentation of conflicting stimuli. Our hypothesis was that CVRT would lead to changes in sensory ratios, specifically increases in the VIS and VEST ratios, suggestive of neuroplastic reweighting towards effective use of residual vestibular senses.

## Methods

### Study design

This prospective, single group, cohort study was conducted in a tertiary otolaryngology clinic. It was approved by the Clinical Research Ethics Board at the University of British Columbia (study # H20-04045) and all experiments were performed in accordance with relevant guidelines, regulations, and the Declaration of Helsinki. The study was performed in a tertiary otolaryngology office in British Columbia, Canada. The study has been registered (Clinicaltrials.gov registration NCT04875013; 04/27/2021). Recruitment took place between April 23, 2021 and June 10, 2021. The treatments were performed between April 29, 2021 and July 23, 2021 and follow up was completed May 16, 2022. All participants provided written informed consent.

### Interventions

Participants completed 12 twice-weekly sessions of CVRT in the clinic. These exercises were designed in accordance with the accepted principles of vestibular rehabilitation to promote compensation (or habituation) and substitution [16, 17]. Participants were challenged to shift their weight along the medio-lateral and antero-posterior axes as directed by an interactive display or to maintain their balance while the support surface moved. During the exercises, participants were challenged to move a cursor that represented their center of gravity towards targets presented on the display or were tasked with shifting their center of gravity to avoid virtual obstacles on the display. Sessions consisted of approximately 10 to 15 different exercises, each lasting one to four minutes for a total session time of approximately 30 min. The exercises grew progressively more difficult over the course of the treatment protocol. The exercise programs were pre-determined and each participant received the same protocol, except to account for the laterality of their deficit.

### Main outcome measures

Consenting participants were invited to the clinic for their baseline assessment where they completed a sensory organization test (SOT) on a computerized dynamic posturography instrument. The SOT test comprises six conditions, each performed in triplicate. The instrument software calculates scores for each condition, as well as a composite score (Table 1). During the posturography tests and all retraining exercises, the participants were supported by a harness as a precaution against falls. The participants also completed three questionnaires: the Dizziness Handicap Inventory (DHI), the Activities-specific Balance Confidence Scale (ABC scale), and the Falls Efficacy Score-International (FES-I). The questionnaire data, and the results of limits of stability test, have been reported elsewhere [14, 15]. These assessments were administered upon enrolment and after completion of the retraining intervention.

**Table 1:** Description of sensory organization test conditions and ratios

Test	Description
Condition 1	Eyes open, fixed visual environment and support surface
Condition 2	Eyes closed, fixed visual environment and support surface
Condition 3	Eyes open, moving visual environment and fixed support surface
Condition 4	Eyes open, fixed visual environment and moving support surface
Condition 5	Eyes closed, fixed visual environment and moving support surface
Condition 6	Eyes open, moving visual environment and moving support surface
SOM	Ratio of scores for conditions 2:1: low score indicates poor somatosensation (e.g., peripheral neuropathy)
VIS	Ratio of scores for conditions 4:1: low score indicates that vision and vestibular senses are insufficient to maintain equilibrium when somatosensory information is unreliable
VEST	Ratio of scores for conditions 5:1: low score indicates that vestibular sense alone is insufficient when vision is absent and somatosensory information is unreliable
PREF	Ratio of the sum of conditions 2,5: sum of conditions 3,6: low score indicates that unreliable visual input is worse than none, suggests visual dependence for postural equilibrium

SOM: somatosensory; VIS: visual; VEST: vestibular; PREF: visual preference

### Data analysis

SOT ratios and changes from baseline are reported as means and 95% confidence interval (95% CI). As an exploratory analysis, participants were stratified according to initial DHI to those with moderate-to-severe disability, (scores > 30) and those with mild disability (DHI ≤ 30) [18]. Post-retraining and follow up results were compared to baseline by mixed-effects analysis with Bonferroni correction for multiple comparisons. Changes in sensory ratios for DHI ≤ 30 and DHI > 30 were compared by two-way ANOVA. We used published normative values for the 50–59 age group [19] as a reference, as this matched the median age of our cohort. This study followed the STROBE guidelines for reporting cohort studies. Analysis was performed using Prism version 10.3.1 (GraphPad Software, San Diego, CA).

### Participants

Candidate participants were identified from patients referred to the primary investigator’s otolaryngology practice: eligible patients were aged between 18 and 80 and reported symptoms of imbalance present for more than six months that negatively affected their day-to-day activities. To be included in the study, the symptoms were clinically assessed to be caused by a stable, non-fluctuating vestibular deficit rather than an active or irritative vestibulopathy based on the criteria of the Barany Society International Classification of Vestibular Disorders (ICVD-1) consensus classification of vestibular symptoms [20]. Objective determination of unilateral peripheral vestibular deficit required at least one of: (a) unilateral weakness during videonystagmography (VNG), as defined by a 25% or greater difference between ears using bithermal caloric testing; (b) significant cervical or ocular vestibular evoked myogenic potential (VEMP) interaural asymmetry, or absent cervical or ocular VEMP responses in one ear with intact responses in the other ear [21]. We excluded individuals who exhibited fluctuating symptoms of an active vestibulopathic cause within the last six months, such as active Menière’s Disease (characterized by fluctuating hearing loss, tinnitus and vertiginous exacerbations lasting > 20 min according to American Academy of Otolaryngology-Head and Neck Surgery criteria); patients with concurrent diagnosis of benign paroxysmal positional vertigo; or patients with clinical and audiometric evidence of a perilymphatic fistula, or otosyphilis. We also excluded those with a deficit that precluded providing informed consent or completing the rehabilitation exercises, such as orthopedic or neurological deficits. Those meeting the eligibility criteria were contacted by telephone and invited to enrol in the study. Enrollment and data collection took place from April 29 2021 to July 23 2021.

## Results

This study enrolled 13 participants with stable unilateral vestibular deficits, which were confirmed by either demonstration of greater than 25% unilateral asymmetry during bithermal videonystagmography (VNG) testing, or a significant interaural ratio difference during cervical or ocular vestibular evoked myogenic potential testing (VEMP). The median age was 51 years (range 18 to 67) and five were female. Seven showed a vestibular deficit by bithermal caloric testing with normal VEMPs, 1 had abnormal cervical VEMP and ocular VEMP but normal videonystagmogram, and 5 had abnormal VEMP and videonystagmogram results (Table 2). All 13 completed the full course of retraining sessions and the post-retraining assessment. Nine participants completed follow up assessment at 4–6 months and 10–12 months.

**Table 2:** Participant demographics and vestibular test results

Median age (range)	51 years (18 to 67)
Number of female / male participants	5 / 8
Previous vestibular rehabilitation	9 of 13 (69%)
Abnormal vestibular test	
VNG	12 of 13 (92%)
oVEMP	6 of 13 (46%)
cVEMP	3 of 12 (25%)

VNG: videonystagmography, oVEMP: ocular vestibular evoked myogenic potential, cVEMP: cervical vestibular evoked myogenic potential

Prior to retraining, the mean SOT composite score was 67.8 (60.2 to 75.5) and this improved significantly and remained above baseline at the 4–6 month and 10–12 month follow up assessments (Table 3). Before CVRT, the participants in this study had a mean SOM ratio of 100.7 (range 0.85 to 1.30), indicating intact ability to use somatosensory information to maintain equilibrium. SOM did not change after CVRT nor during follow up assessments. The initial VIS and VEST ratios indicated significant difficulty maintaining equilibrium on the moving, sway-referenced platform prior to retraining. After CVRT, VIS increased significantly above baseline and remained elevated during 4–6 and 10–12 month follow up. VEST, likewise, increased after CVRT and was significantly above baseline at the 10–12 month timepoint, though the 4–6 month assessment was not significantly different from baseline. The PREF ratio was 93.3 (86.4 to 100.2) prior to CVRT and changed negligibly after retraining and during follow up.

**Table 3:** Baseline sensory ratio scores and changes after computerized vestibular retraining therapy

	Ratio at	Change in ratio		
	Baseline	Post-CVRT	4–6 months	10–12 months
**Sensory Organization Test**				
Composite score	67.8 (60.2 to 75.5)	**11.4 (5.0 to 17.9)**	**8.8 (1.5 to 16.1)**	**1.5 (3.2 to 17.8)**
**Ratio**				
SOM Condition 2:Condition 1	100.7 (94.6 to 106.7)	-3.0 (-9.5 to 2.5)	-0.3 (-7.0 to 6.4)	-1.0 (-7.6 to 5.7)
VIS Condition 4:Condition 1	76.6 (65.1 to 88.1)	**11.6 (1.6 to 21.7)**	**11.9 (0.6 to 23.2)**	**12.1 (0.8 to 23.4)**
VEST Condition 5:Condition 1	68.4 (58.1 to 78.8)	**9.5 (0.6 to 18.3)**	7.5 (-2.6 to 17.5)	**12.0 (2.0 to 22.1)**
PREF Σ (Conditions 3,6): Σ (Conditions 2,5)	93.3 (86.4 to 100.2)	5.5 (-2.1 to 13.0)	2.7 (-5.6 to 11.1)	1.3 (-7.1 to 9.6)

SOM: somatosensory; VIS: visual; VEST: vestibular; PREF: visual preference

**Fig. 1:**
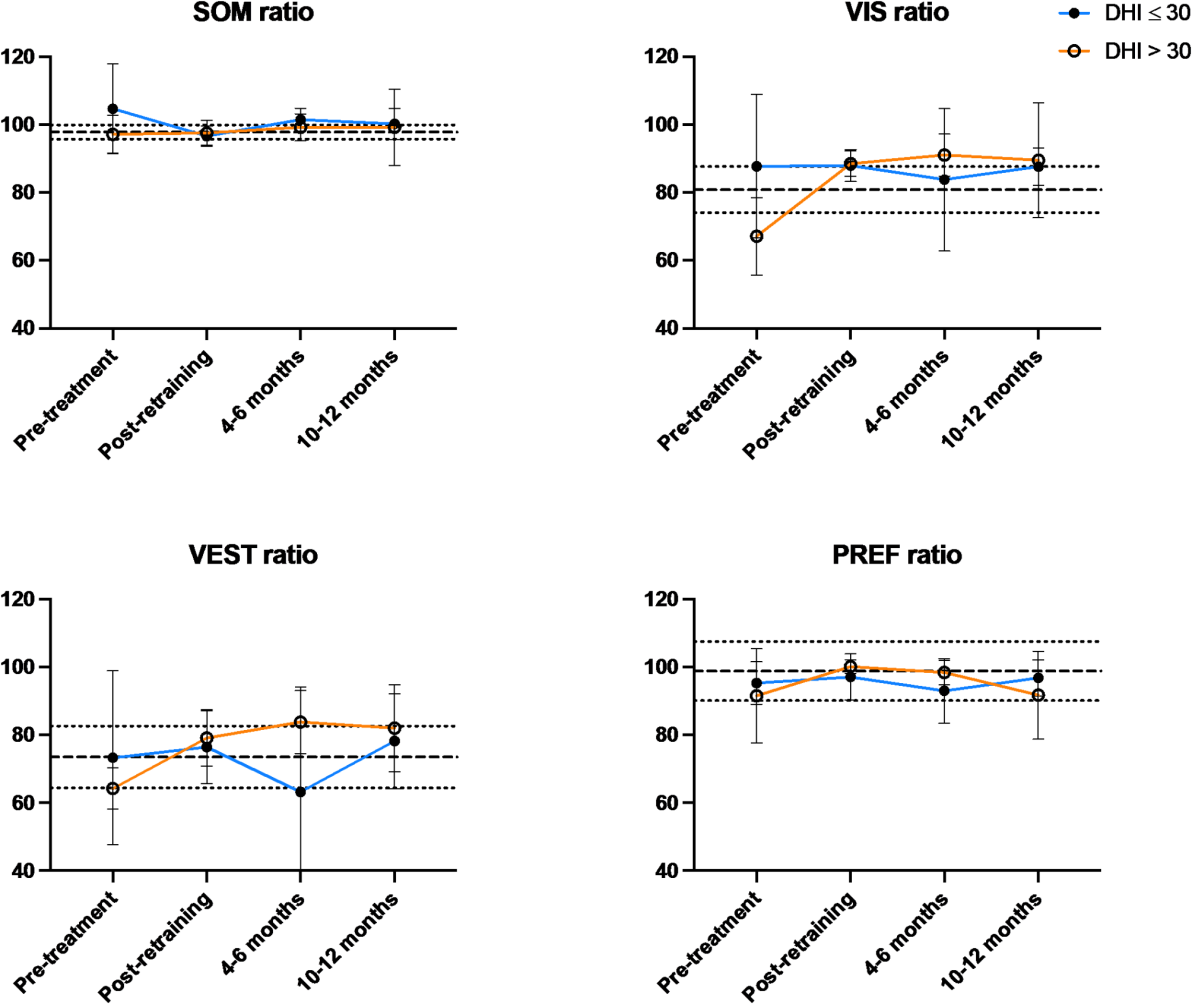
SOT sensory ratios for DHI ≤ 30 (blue line; *n* = 6) and DHI > 30 (orange line; *n* = 7) at baseline, immediately post-retraining, and at 4–6 months and 10–12 months after retraining. The heavy dashed line is the normative mean and the light dotted lines are +/- one SD from the mean.^1^

To determine whether outcome was associated with symptom severity prior to retraining, we stratified the cohort to participants with low DHI (≤ 30) and those with high DHI (> 30). We found that, at baseline, the low DHI group scored close to published age-matched normative values for all four ratios (compared to healthy individuals aged 50–59 [19]), while the high DHI group had VIS and VEST ratios below normative means (Fig. 1).

After retraining, in the high DHI group, VIS increased by a mean of 21.4 (4.5 to 38.3) and VEST improved by 14.9 (3.3 to 26.5) after CVRT while the SOM and PREF ratios did not change. No changes were observed in the low DHI group. Analyzing changes from baseline for the three post-treatment assessments in aggregate, there were no differences for SOM and PREF between groups; however, the increase in VIS (*P* = 0.0006) and VEST (*P* = 0.02) was significantly higher in the high DHI group.

## Discussion

**Fig. 2:**
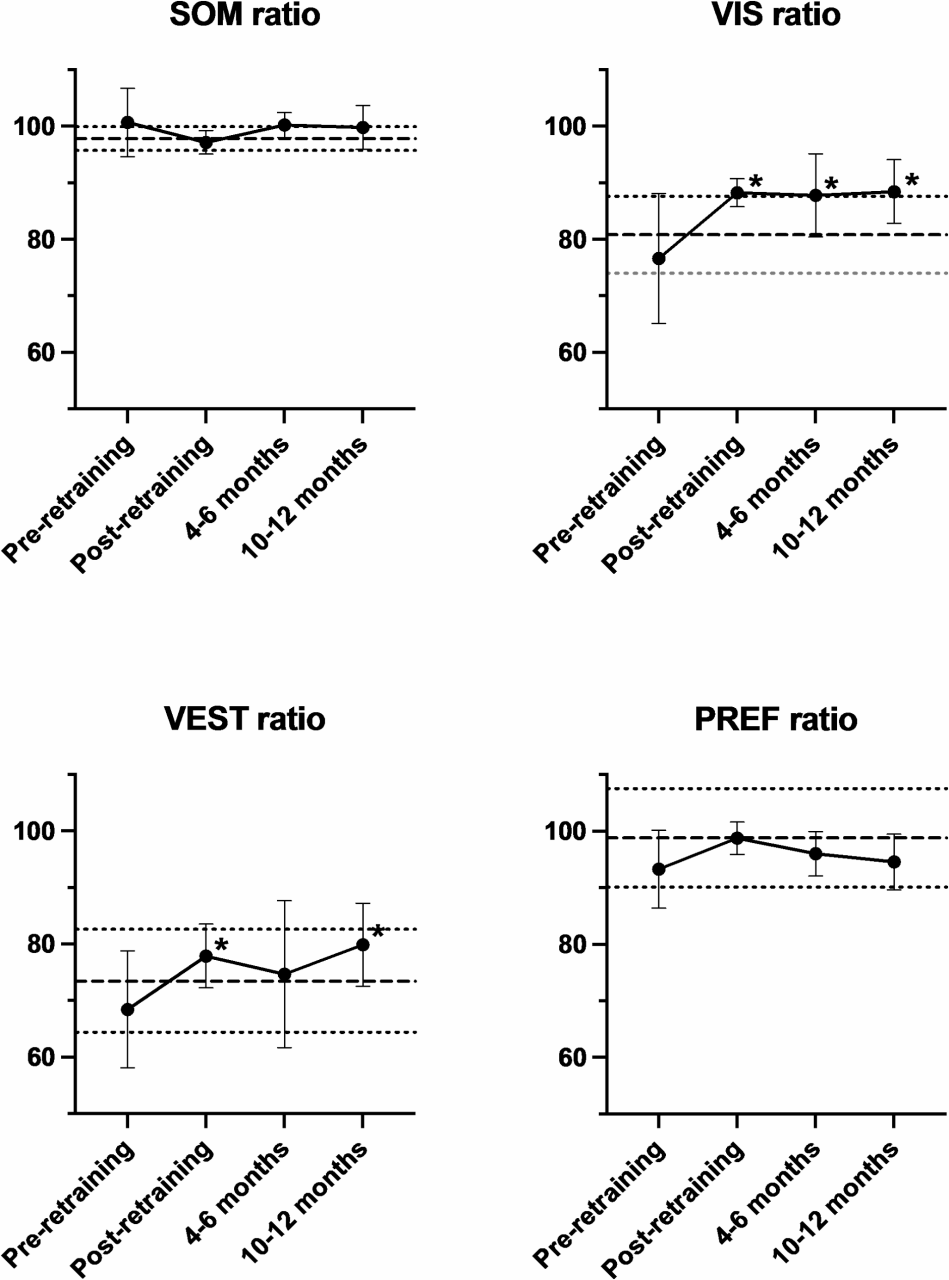
SOT sensory ratios at baseline, immediately post-retraining, and at 4–6 months and 10–12 months after retraining. Asterisks indicate improvement compared to baseline (*P* < 0.05). The heavy dashed line is the normative mean and the light dotted lines are +/- one SD from the mean.^1^

This study used the SOT test, an objective posturography assessment, to measure changes after CVRT in postural control of patients with symptoms lasting greater than 6 months. The participants in this study had stable symptoms for greater than six months and had achieved static compensation; accordingly, they all had SOM ratios near 1. The VIS and VEST ratios both incorporate testing on the sway-referenced platform, which presents a significant challenge to postural control. Indeed, balance scores on the sway referenced platform were significantly lower than on the fixed platform, both for healthy participants [22] and for those with vestibular deficits [19]. After CVRT, participants displayed improved postural stability on the sway referenced platform, both in the presence of visual cues (VIS) and in their absence (VEST). There was no change in PREF, suggesting no strong visual dependence (Table 3; Fig. 2). Collectively, these changes associated with CVRT, independent of visual cues, suggest that improved postural stability arose from a gain in vestibular function.

In the days after unilateral vestibular injury, the asymmetry in neural activity of the vestibular nuclei is modulated to restore homeostasis. This coincides with acute compensation, whereby spontaneous nystagmus is resolved and static balance symptoms are greatly ameliorated. Improvements in dynamic balance, through complex and diverse electrophysiologic and behavioral changes, continue to take place for most individuals over the ensuing weeks; however, many individuals continue to experience significant morbidity months or years after onset, even with treatment [6].

Horak found that well-compensated patients had lower vestibulo-ocular reflex gains than poorly compensated patients and suggested that residual, possibly distorted vestibular information was worse than none at all; however, those who learned to use remaining vestibular information from the intact ear performed better than those who relied heavily on visual and somatosensory cues [12]. Individuals with vestibular deficits may adopt diverse motor learning strategies navigate the requirements of daily life [23] and these strategies may place a higher or lower dependence on vision [24].

Consistent with this literature, we observed significant inter-individual variability in our participants prior to CVRT. The changes we observed after CVRT, namely improved global balance performance even in the absence of a somatosensory reference and of visual cues, coupled with the reduction in the between-participant variability, suggest that that compensation by use of remaining vestibular function, as described by Horak, was taking place for the participants in this study.

### Vestibular rehabilitation modalities

One consequence of continuous and evolving compensation is that early compensatory changes take place while neurological healing and restructuring is ongoing. Tighilet and Chabbert wondered, in their 2019 review, how sensory input from re-afferentiated vestibular organs would be re-integrated and whether repaired synapses that do not exactly recapitulate the pre-injury state could lead to aberrant sensory input during movement [2]. For some patients, early compensation strategies that are ‘good enough’ may fail to reintegrate retained or restored vestibular function into their postural maintenance strategy. Training protocols that call upon vestibular input may promote reweighting from an over-reliance on vision and somatosensation to a more balanced integration of sensory information that makes use of vestibular senses retained by the patient. However, common treatment modalities may not elicit such balanced compensatory responses [11, 25].

Most rehabilitation modalities seek to recalibrate postural (and ocular) control to habituate to vestibular information that is absent, corrupted, or asymmetric. Through coordinated head, body, and eye movements, patients are trained to cope with their vestibular deficit and maintain postural control primarily through the visual and somatosensory cues [12].

Virtual reality-based interventions have attracted the attention of researchers and clinicians; however, there is conflicting evidence concerning whether such interventions are superior to conventional vestibular exercises [26–29]. Many of these interventions were designed as more engaging alternatives to conventional vestibular exercises [27, 30]; however, most share with conventional exercises that they are designed to promote habituation and adaptation by encouraging head and eye movements in conjunction with concordant visual information. It is less clear that these types of exercises help with dynamic balance and improve capacity to manage discordant sensory information; indeed, some forms of visual stimuli may even exacerbate visual dependence [11].

There is some evidence in the literature that training that incorporates incongruent sensory information– that is, when visual, somatosensory, and vestibular cues are not in agreement– may help improve dynamic balance. For instance, training using tilting platforms [10, 31] prompts patients to maintain their balance in a dynamic environment in which somatosensory input from their feet and ankles in unreliable.

Compensation achieved through suppression of vestibular information, as occurs in the acute phase of vestibular injury and as evidenced by atrophy of parts of the brain involved in vestibular processing [32, 33], may limit the potential for dynamic recovery when vestibular loss is partial or temporary. CVRT seeks to train patients to use their remaining vestibular function, either in conjunction with congruent visual and somatosensory information, or to overcome incongruent visual and somatosensory cues, in a manner that more closely replicates the integration of sensation by individuals with no deficit. By challenging participants to maintain their balance on an unsteady surface and in visually complex environments, participants in this study improved their postural control in in a variety of conditions.

The capacity of the vestibular system– both central and peripheral components– to change, regenerate, and repair, help to explain the mechanisms by which many individuals recover high levels of function after vestibular loss. Such research also offers insight on how individuals that do not achieve robust dynamic compensation, either spontaneously or through standard vestibular therapy, may benefit from retraining. In this pilot study, CVRT was associated with improved postural control consistent with increased weighting of information from the vestibular organs– either on the unaffected side or from intact organs on the affected side– over vision through a mechanism of vestibular plasticity.

## Conclusions

CVRT is associated with durable improvement in global balance and changes in the way sensory information is weighted to achieve postural control, in particular when vision and somatosensation are unreliable or absent. Changes were consistent with increased weighting of vestibular information over vision.

### Limitations

This single-group study did not include a no treatment or alternative treatment control and enrolled a small sample size of 13 participants. No sample size calculation was performed *a priori.* We enrolled participants with persistent, stable symptoms in an effort to minimize variability; however, we cannot rule out symptom variability unrelated to treatment. Individuals with mild impairment showed no benefit but we cannot determine whether this was because of a ceiling effect of the SOT or whether those with mild impairment do not respond to treatment. Improvement in SOT scores due to learned familiarity with the test [34] is a potential source of bias.

## Data Availability

The dataset generated during the current study is not publicly available to protect the privacy of participants. Following publication, the data that underlie the results reported in this article are available from the corresponding author to researchers who provide a methodologically sound proposal and sign a data access agreement, the conditions of which protect participant confidentiality.

## Abbreviations

ABC: Activities-specific balance confidence scale
CDP: Computerized dynamic posturography
CVRT: Computerized vestibular retraining therapy
DHI: Dizziness handicap inventory
FES-I: Falls efficacy score - international
LOS: Limits of stability test
PPPD: Persistent postural-perceptual dizziness
SOT: Sensory organization test
VEMP: Vestibular evoked myogenic potential
VNG: Videonystagmography
VOR: Vestibular ocular reflex
